# Barriers and facilitators to pre-exposure prophylaxis (PrEP) eligibility screening and ongoing HIV testing among target populations in Bondo and Rarieda, Kenya: Results of a consultation with community stakeholders

**DOI:** 10.1186/1472-6963-14-231

**Published:** 2014-05-21

**Authors:** Natasha Mack, Jacob Odhiambo, Christina M Wong, Kawango Agot

**Affiliations:** 1 Social and Behavioral Health Sciences, Durham, NC FHI 360, USA; 2 Impact Research and Development Organization, Kisumu, Kenya

**Keywords:** Pre-exposure prophylaxis, PrEP, HIV, PrEP implementation, Sub-Saharan Africa

## Abstract

**Background:**

As pre-exposure prophylaxis (PrEP) moves closer to availability in developing countries, practical considerations for implementation become important. We conducted a consultation with district-level community stakeholders experienced in HIV-prevention interventions with at-risk populations in Bondo and Rarieda, Kenya to generate locally grounded approaches to the future rollout of oral PrEP to four populations: fishermen, widows, female sex workers, and serodiscordant couples.

**Methods:**

The 20 consultation participants represented the Ministry of Health, faith- and community-based organizations, health facilities, community groups, and nongovernmental organizations. Participants divided into breakout groups and followed a structured discussion guide asking them to identify barriers to implementing HIV-prevention interventions (including PrEP) with each population. Questions also solicited solutions for addressing these barriers, as well as other facilitators for PrEP implementation. In particular, questions focused on how to encourage people to screen for PrEP eligibility by having HIV and other blood tests and how to encourage compliance with ongoing HIV testing.

**Results:**

The barriers and facilitators/solutions discussants provided were frequently population-specific, but there were also broad-level similarities across populations. Service delivery barriers to HIV-prevention interventions concerned the need for staff trained to address the needs of particular populations. Service delivery facilitators to provision of ongoing HIV testing consisted of offering testing options besides facility-based testing. Stigma was the main community-level barrier for all groups, whereas barriers at the level of target populations included mobility; lifestyle and life circumstances, especially cultural norms among fishermen and widows; and fears, lack of awareness, and misinformation. Proposed facilitators and strategies for addressing community- and population-level barriers included topic-specific education within the populations and community, involvement of partners and family members, mass HIV testing, and peer educators. Barriers to PrEP uptake included non-adherence to pill taking and missing clinic visits. For drug adherence, facilitators were counselling and involving family members. Discussants suggested that client reminders, e.g., home visits, were needed to encourage clients to keep their clinic appointments.

**Conclusions:**

Strategies for encouraging eligibility screening and ongoing HIV testing will have local and population-specific aspects. Our results nonetheless apply to similar populations throughout sub-Saharan Africa and reach beyond oral PrEP to other ARV-based PrEP formulations.

## Background

As pre-exposure prophylaxis (PrEP) moves closer to potential availability in developing countries, practical considerations for its implementation become ever more important. Guidance on PrEP implementation for the antiretroviral drug Truvada (emtricitabine and tenofovir disoproxil fumarate, or FTC/TDF) is becoming available, beginning with clinical recommendations from the U.S. Centers for Disease Control and Prevention (CDC) for the U.S. context [1-3] and from the World Health Organization (WHO) for a global audience [4]. These recommendations for clinicians address client eligibility, the prescribing of FTC/TDF as PrEP, testing schedules for HIV infection and liver and renal function, follow-up schedules for provider visits and pill refills, and considerations for discontinuing PrEP. The populations these guidelines address include men who have sex with men, transgender women who have sex with men, heterosexually active men and women, serodiscordant couples, and injecting drug users.

Other kinds of practical guidance also are needed, such as for integrating PrEP into existing service delivery settings and providing community education about PrEP. However, much of this guidance must necessarily be developed at a micro-level in local country and community contexts, as will be the case for behavioural components of PrEP interventions [5]. Such locally developed strategies must also build on the knowledge and experience gained from current and past interventions with the target populations.

As part of an ancillary study of the FEM-PrEP clinical trial that tested once-daily FTC/TDF as PrEP for HIV prevention among women at risk of HIV infection [6], we conducted a consultation with district-level community stakeholders experienced in HIV-prevention interventions with populations at risk of HIV in Bondo and Rarieda districts, Nyanza province, Kenya. Our aim was to answer the following research question: What are some locally grounded approaches to future rollout of oral PrEP to local populations at risk of HIV infection, if clinical trials show the method to be effective? More specifically, based on lessons learned from the provision of HIV-prevention interventions, what might be some of the barriers, solutions, and facilitators related to the clinical screening of potential clients for eligibility to take oral PrEP, and to ongoing HIV testing for PrEP users in four local target populations (fishermen, widows, female sex workers, and serodiscordant couples)? The results reported here are intended to inform the design of future programs geared toward the four target populations in Kenya, as well as other similar populations in the sub-Saharan African region.

## Methods

This consultation was conducted with stakeholders from Bondo and Rarieda districts, rural communities bordering Lake Victoria in Nyanza province, Kenya. Given that medical male circumcision has been shown to reduce the incidence of HIV infection [7,8], one current HIV-prevention strategy in the province consists of efforts to provide voluntary medical male circumcision to men in the Luo ethnic group, in particular. In 2008 to 2009, only 45% of men in Nyanza—and 22% of men in the Luo ethnic group—were circumcised, compared to at least 90% in all other provinces [9]. Other HIV-prevention efforts in the region include the communication of behaviourally-focused messages that advise people to use condoms and limit their number of partners/be faithful to one partner, encourage young people to practice abstinence or delay sexual debut [9], and promote HIV testing and treatment; the prevention of mother to child transmission; prevention with positives; and condom distribution. In 2008 to 2009, the overall prevalence of HIV in Nyanza was 14% (16% among women and 11% among men), whereas in Kenya as a whole the prevalence was 6% (8% among women and 4% among men) [9].

FHI 360, the sponsor institution, collaborated on this study with Impact Research and Development Organization (Impact RDO), which also implemented the FEM-PrEP clinical trial with which our study was associated at the Bondo, Kenya site. The local research team conducted all data collection in a culturally appropriate manner through the use of consultation methodology—a participatory approach that acknowledges participants as experts in their field rather than viewing them as research “subjects.” This approach also helps to create a sense of local ownership over the problem-solving process.

### Ethics review

The consultation was approved as part of a larger study entitled *Sociobehavioral Research and Community Planning to Develop Site-Specific Pilot Intervention Plans for PrEP Rollout* by the Protection of Human Subjects Committee at FHI 360 in December 2008 and from Kenyatta National Hospital Ethics and Research Committee (KNH-ERC) in April 2009. The ethics committees did not consider the consultation component of the study to be human subjects research; therefore, the consultation participants were not required to provide their informed consent. Nonetheless, they were aware that they were contributing to a research study whose results would be disseminated. Data collection was stopped before the study was completed, when an independent data monitoring committee found that FEM-PrEP would be unable to demonstrate the effectiveness of FTC/TDF as oral PrEP. Both the FEM-PrEP clinical trial and the study reported on here were then ended in 2011.

### Consultation description

The objectives for the consultation were:

• To identify barriers, challenges, and facilitators to implementing oral PrEP with local target groups

• To develop strategies or solutions to address each barrier and challenge, particularly in the following areas:

o Educating the target group about daily oral PrEP (data outside the scope of this paper)

o Recruiting the target group for clinical screening tests to see if they qualified to take daily oral PrEP

o Encouraging compliance with quarterly HIV tests for ongoing monitoring

We selected four target populations on whom to focus during the consultation activities—two broad groups and two specific groups, elaborated below. This selection was based on populations identified as local priorities for oral PrEP during semi-structured interviews with civil society leaders and public health stakeholders. These four populations were:

1. Young married and unmarried men, including fishermen and motorcycle/bicycle transporters

2. Young married and unmarried women, including women at the beaches of Lake Victoria and Luo widows

3. Female sex workers

4. HIV serodiscordant couples

The first two groups were to be narrowed down into smaller sub-groups by the consultation participants, because we believed that the issues around HIV prevention and PrEP implementation might be distinct for different sub-populations of these broader groups.

### Consultation participants

Consultation participants consisted of 20 individuals from Bondo and Rarieda who were experienced in working with at-risk populations. Organizations that were represented included the Ministry of Health, faith- and community-based organizations, health facilities, community groups, and nongovernmental organizations. If the study staff did not know of anyone at a given organization who was qualified to participate in the consultation, personnel at the organization identified an appropriate individual. Each invited participant was asked to indicate his or her first and second choice of the four target populations to discuss during breakout groups. We then assigned consultation participants to four groups of five discussants each based on their experience and preferences. We limited the number of people invited to the consultation to 20. This was to ensure that the breakout groups were small enough to encourage active participation by everyone in the groups.

### Breakout group discussions

For the breakout groups, the FHI 360 and Impact RDO researchers co-developed a structured discussion guide. Questions focused on:

• Challenges of working with this target group in the context of HIV prevention

• Successful and unsuccessful strategies for influencing this group

• Possible barriers/challenges organizations might face in providing PrEP to the target group, as well as strategies for addressing them

• Strategies for educating the target group (both in groups and individually) about PrEP

• Barriers/challenges organizations would face in recruiting members of the target group to come to a health clinic for blood (HIV and liver/kidney function) and urine (for pregnancy) screening tests to learn if they qualified to take PrEP, and strategies for addressing them

• Barriers/challenges related to HIV testing as part of eligibility screening, and strategies for addressing them

• Strategies for improving HIV testing services in order to test people in each target group every 3 months

The discussion guide included tables for participants to complete to help them systematically record the barriers/challenges and solutions/facilitators generated during the breakout group discussions.

### Consultation

The consultation was held on June 30, 2010 at a hotel in Bondo. The agenda consisted of sessions that described PrEP in general;^a^ the FEM-PrEP clinical trial; and the *Sociobehavioral Research and Community Planning* study, including results of the data collected [10,11]. These presentations provided participants with a context for discussions about rolling out PrEP to the different potential target populations. The sessions were followed by question-and-answer periods and group discussion.

Consultation participants then divided into their assigned breakout groups, responded to the questions in the guide during group discussion, and completed the tables provided. A note taker for each group also recorded the main points on a flip chart. At the end of the breakout period, each group reported the highlights of their discussion back to the larger group. A wrap-up session was held before the consultation was adjourned. We collected the completed question guides and flip chart notes in order to aggregate the completed tables for distribution to attendees. Results were also later shared in subsequent consultations with public health stakeholders at the provincial and national levels.

### Data analysis

A researcher from FHI 360 then analysed the data iteratively. The analyst used Nvivo 9.0, a qualitative data analysis software, to code the information in the aggregate tables as pertaining to one of the four target populations. The analyst then further coded the data as either a “barrier” or a “solution/facilitator,” using emergent (i.e., not pre-defined) sub-codes. For example, a segment of the data might be coded as “serodiscordant couples,” and then also have the code “Barrier – Stigma” applied. Individual “barrier” and “solution/facilitator” sub-codes were then assessed to see whether they were related to each other through any common theme. The analyst then determined that all sub-codes could be grouped into one of three themes: 1) service delivery-level barriers and facilitators/solutions, 2) community-level barriers and facilitators/solutions, and 3) target population-level barriers and facilitators/solutions. The researchers who collected the data then verified the data interpretation and corrected inaccurate assumptions.

### Rationale for the consultation format

We chose the day-long consultation format to collect this data for two reasons. First, before collecting information from the consultation participants, we needed to inform them about oral PrEP in general, the FEM-PrEP clinical trial, and the ancillary research study at hand—all of which would together take more time than a focus group or interview would allow. Second, we believed that we would gain the most information from an interactive approach in which consultation participants worked together to apply previous lessons learned as they jointly developed locally grounded strategies.

### Description of target populations

The breakout groups focusing on young men and women elected to focus on fishermen and widows, respectively, based on their unique life circumstances and high-risk sexual behaviours. The four target populations discussed in the consultation were thus fishermen, widows, female sex workers, and HIV serodiscordant couples. We will first describe each group briefly before presenting the consultation results.

#### Fishermen

Fishermen who work on Lake Victoria are at high risk of HIV infection due to having multiple partners with whom they often have unprotected sex. These partners may include wives, girlfriends, and female fish traders [12]. Fish populations are dispersed in Lake Victoria, such that fishermen must be mobile in order to be successful. The lack of refrigerators at the beaches means that fish have to be sold immediately, and as a result, fishermen establish multiple home bases and families in the landing communities closest to the fish populations [13]. In addition, the ratio of fishermen to female fish traders can be as high as one to three, creating competition among women for the right to buy fish to sell in the market. This gives fishermen leverage to make sexual demands of fish traders, such as unprotected sex [13]. Fishermen may also have unprotected sex because of a culture of risk denial or risk confrontation related to fishing as a high-risk occupation [14]. Fishermen’s subculture has also been characterized as including tolerance of danger, fatalism, and the need to prove their masculinity [15]. As Mojola [13] notes, “[T]his orientation, along with heightened uncertainty of life expectance, and cultural norms supporting living life to the fullest might partly account for why unprotected sexual relationships persisted in the face of growing AIDS related mortality.” Other risk factors for HIV among fishermen include alcohol use as a coping mechanism for job stress; extended time spent away from home, leading to multiple sex partners; high mobility; the young age of people in this occupation; access to disposable cash on a daily basis; availability of commercial sex in fishing ports; and social marginalization [12,14].

A 2005 survey of 250 fishermen in Kisumu, Kenya found them to be young (half were under 26 years old) and married, with 14% in polygamous marriages. Nearly all of the married men were involved in extramarital sex characterized by low condom use. Two-thirds of the fishermen surveyed reported engaging in transactional sex—providing money, shoes, clothing, and the right for female fish traders to buy his fish in exchange for sex [16].

#### Widows

Traditionally, widows in the Luo ethnic group, the predominant ethnic group in Nyanza province, are inherited by a male relative of the deceased husband. Unprotected sexual intercourse is required for sexual cleansing of the widow as part of inheritance rituals, as well as during rituals preceding major events, such as before planting or harvesting. However, relatives of the deceased husband are increasingly hiring non-relative professional inheritors or cleansers due to the fear of HIV, as many women’s husbands are feared or known to have died of AIDS [17-21]. Both professional inheritors/cleansers and inheritors related to the deceased husband typically have multiple sex partners (e.g., their wives and potentially other widows), increasing the risk of HIV transmission.

Social pressures within the community encourage widows to be inherited. For example, they otherwise may not be permitted to enter homesteads or work, they are prohibited from and impede other family members from planting, and misfortune is said to be likely to befall their children [19,21]. Widows often consider inheritance beneficial because it allows them to gain social acceptance within the community, prevents them from having multiple sexual partners, enables them to conceive more children, can be economically beneficial, and allows for satisfaction of sexual needs [21]. However, whereas traditionally widow inheritance meant economic and social support for the widow, many inheritors are reported to no longer contribute financially to the household and may in fact expect to be provided with food and services. Furthermore, children fathered by the inheritor are believed to belong to the deceased husband and are often not supported financially by their biological father [18,21].

Uninherited widows are also at risk of HIV infection—they are often in dire economic circumstances and may have unprotected sex with multiple men who might help them economically. They may not use condoms with boyfriends, who are often older, married, and perceived as less risky than inheritors [22].

Agot and colleagues found that widows inherited by non-relative males for sexual rituals were more likely to be infected with HIV than those not inherited, and that widows inherited for companionship by relative males were less likely to be infected than uninherited widows [17]. Luke [22] found that inherited widows are more likely than married women and uninherited women to believe that they are at higher risk of HIV infection; as a result, some widows elect not to be inherited [22]. In Agot et al.’s sample of 1,987 Kenyan widows, the women had a mean age of 35 years, and nearly all had children [17].

#### Female sex workers

Female sex workers accounted for 14.1% of the new HIV infections in Kenya in 2008 [23]. Voeten’s [24] description of self-identified female sex workers in Saida and Bondo districts of Nyanza indicated that they tend to be in their early to mid-twenties, are unmarried (more than half are separated or divorced), and have children who depend on them economically. In addition to the sex partners they consider to be clients, women also tend to have at least two regular sex partners — including some former clients — whom they do not consider to be clients and who may possibly pay them for sex directly or indirectly (e.g., rent, food, clothing, or school fees). As in other sex worker populations, consistent condom use is lower with regular partners (about 25%) than it is with clients (about 60%) [24].

These women’s risk factors for HIV infection include having multiple concurrent partners who often have multiple partners themselves [12], drug and alcohol abuse leading to high-risk behaviours [12,25], and anal sex as a result of client demand [26]. Sex without condoms may be more highly remunerated than protected sex [27], and other types of work may pay less [12]. On the other hand, sex workers in Kenya and other locations in sub-Saharan Africa have also been found to use condoms more frequently with clients than they do with boyfriends or primary partners, but they may use condoms less frequently with regular clients due to a perception that HIV risk is low with regular partners in general [12,28-30]. Another factor contributing to sex workers’ vulnerability to HIV is the illegal status and clandestine nature of sex work in Kenya. As a result, women may not admit to doing sex work, making it difficult to reach them with interventions [12].

#### HIV serodiscordant couples

In 2007, the Kenya AIDS Indicator Survey found that among the nearly 18,000 couples surveyed, 10% had one or both partners infected with HIV and 60% of those couples were serodiscordant. An estimated 344,000 couples were then said to be serodiscordant across Kenya [31]. In Nyanza Province, 13% of couples were serodiscordant in 2008–2009 [9]. Serodiscordant couples were identified as an at-risk group in the Kenya National AIDS Strategic Plan [23], but prevention strategies have been found to be weakly implemented, contain no systematic messages, and have been left to research pilot projects [32].

Demographic and behavioral characteristics of discordant couples were reported for the Partners PrEP study, a randomized clinical trial of daily oral TDF and FTC/TDF PrEP to decrease HIV-1 acquisition within HIV-1 serodiscordant heterosexual couples that included four sites in Kenya. In that study, the median age of HIV-negative partners was 33 years, and 62% of those partners were male. Most couples were married, for a median of 7 years. Twenty-seven percent reported unprotected sex during the month prior to enrollment, and sex with outside partners was reported among 14% of HIV-negative male and 1% of HIV-negative female partners [33].

The negative partner’s vulnerability to HIV infection includes unprotected sex, the positive partner’s fear of disclosing their HIV-positive status to the uninfected partner due to concerns about stigma, and a belief that serodiscordance within couples is not possible [34]. In one study in Rift Valley, Kenya, HIV-positive women in discordant couples reported fearing that if their status was seen as indicative of infidelity, their children could be disinherited [34]. In the Kenya AIDS Indicator Survey 2007, members of married or cohabitating couples were found to be more likely to disclose their status to their partner (86.2% of women and 76.4% of men) than were people with girlfriends/boyfriends or in casual relationships [31].

## Results

Below, we present the barriers and facilitators/solutions to PrEP introduction that were identified during our regional consultation.

### Service delivery-level barriers and facilitators/solutions

Several barriers and facilitators were identified related to service delivery for HIV prevention in general. One such barrier for serodiscordant couples was the dearth of health workers at the local level who were trained on issues relevant to serodiscordant couples (e.g., the stigma and discrimination that serodiscordant couples face; the need to provide couple counselling to help them understand discordant status and how to avoid HIV transmission; and the psychological issues that couples face when one partner is HIV positive and one negative, potentially leading to a breakdown in their relationship). For female sex workers, the stakeholders described the inaccessibility of female condoms—a female-controlled method of HIV prevention—as a barrier to health educators being able to advocate for women to protect themselves against HIV. They said that female condoms are both expensive and difficult to find.

For PrEP provision specifically, discussants focusing on widows highlighted human resources as a barrier. They said that there were not enough trained staff who could provide mentoring to junior staff, whose capacity would need to be improved in order to assist with PrEP service provision and with conducting follow-up activities for widows taking PrEP. Stakeholders discussing serodiscordant couples cited the lack of equipment in laboratories as a barrier for both PrEP provision and HIV testing for PrEP users. They said that this could be addressed by encouraging cost-sharing and collaboration among stakeholders in order to hasten the process of testing blood samples. Couple counselling and partner referrals were suggested as facilitators for HIV testing for fishermen and serodiscordant couples. Consultation participants suggested providing mobile HIV testing and counselling to fishermen as a way to improve provision of regular, ongoing HIV testing for PrEP users, whereas providing ongoing home-based HIV testing and counselling was suggested for all other target groups. “Moonlight VCT” (referring to voluntary counselling and testing [VCT] services that are offered at night) along the beaches for fishermen and staffed by peer educators for female sex workers was also suggested. In addition, the widows discussion group suggested deploying enough counsellors to community VCT sites to improve service provision. They also suggested providing widows with incentives (e.g., whether monetary incentives, services, or material goods) for HIV testing. Discussants in the female sex worker and serodiscordant couple groups suggested establishing effective follow-up systems to help PrEP users adhere to clinic visits and regular HIV tests. They mentioned client reminder systems like using schedule cards, home visits, and phone calls to reduce defaulters.

### Community-level barriers and facilitators/solutions

Stakeholders suggested potential resistance to PrEP on the part of the community and family members as a barrier to PrEP implementation among fishermen. They recommended providing education about PrEP to the general community, family members, and sexual partners. Similarly, the consultation discussants cited the community’s potential non-acceptance of PrEP as a barrier to implementing the method among serodiscordant couples; a solution would be to work with community stakeholders and gatekeepers to conduct social marketing.

Stigma was cited as a barrier to successful HIV prevention interventions among all target populations. Consultation participants cited the stigma of getting tested for HIV as making it difficult to convince fishermen and female sex workers to access HIV testing for PrEP eligibility screening and ongoing PrEP monitoring. For fishermen, mass HIV testing among the general population, moonlight VCT, and community education were suggested as strategies to address the stigma, whereas community education, advocacy, and peer educators were suggested to address any stigma affecting female sex workers’ willingness to get tested for HIV.

Stigma associated with the testing of blood samples for liver and kidney function during clinical screening for PrEP eligibility was mentioned for fishermen, widows, and serodiscordant couples; participants proposed addressing such stigma by conducting community education about blood draws.

Discussants in the breakout groups for both fishermen and widows anticipated that there would be stigma in the community related to the fact that FTC/TDF (the antiretroviral drug that was being tested in FEM-PrEP and was subsequently found partially effective as PrEP in other trials) is also used to treat people who are infected with HIV. They suggested continuous education to debunk myths about HIV, involvement of female partners (of fishermen), and community sensitization to encourage people to accept those taking FTC/TDF as PrEP.

Stigma by the community and families directed against people who are HIV positive was said to make it difficult to provide HIV-related services (including PrEP in the future) to serodiscordant couples. The stigma associated with disclosure was also described as problematic for this group. Solutions that discussants proposed consisted of providing continuous community education with the goal of minimizing stigma and educating family members and partners to accept people living with HIV.

### Target population-level barriers and facilitators/solutions

#### Access to target populations

Discussants mentioned that gaining access to the target populations was problematic because of their occupational mobility (as described earlier) and night time working hours. Discussants said that in addition, sex workers do not like to be identified as such because sex work is illegal in Kenya; when sex workers are subjected to sexual violence, they said, it can be challenging to successfully encourage them to seek legal redress.

Serodiscordant couples were described as a hard-to-reach population because they do not like to disclose their HIV status publicly due to stigma. Also, the high rate of divorce among serodiscordant couples was discussed as leading at least one member of the couple to relocate to a different home, making him or her difficult to locate. The discussants suggested that the potential for divorce could be addressed by training health workers and counsellors to engage with couples, and by conducting continuous couples counselling to help couples understand how to avoid HIV transmission to the negative partner. In addition, discussants said that widows and members of other groups who relocate after starting PrEP must be targeted with education on the importance of reporting relocation.

#### Lifestyle and circumstances of target populations

The culture of fishermen—the tendency to have multiple, often concurrent, sexual partners—and fishermen’s negative attitudes towards HIV-prevention information were cited as challenges to any HIV-prevention intervention. Discussants indicated that discussion groups and seminars that promote HIV/AIDS awareness, as well as recreational activities like sports and community drama, have been successful in engaging fishermen and reaching them with HIV-prevention information and services.

The cultural traditions of the Luo ethnic group were described as negatively affecting the ability of Luo widows to access and use HIV prevention services and methods. The group cited specifically the cultural practice of widow cleansing that prohibits women from using condoms during sexual cleansing rituals. A successful strategy for working with widows on HIV prevention has been to involve them directly in planning activities, allowing them to “own” programs. Community meetings were suggested to educate them on their human rights related to HIV prevention. The group discussing widows also suggested encouraging them to form groups in which they could initiate income-generating activities to boost their financial status and reduce their economic deprivation and dependency.

Similarly, the consultation participants described that in their experience, workshops on human rights, legal issues, and risk reduction methods have been successful in empowering female sex workers to minimize their risk of HIV infection, along with income generation activities and microfinance opportunities to help them give up sex work and reduce their risk.

Discussants in the female sex worker and serodiscordant couple groups noted support systems for PrEP users as a facilitator for PrEP use and HIV-prevention interventions. In particular, they recommended support groups and prevention-with-positives activities where serodiscordant couples could obtain peer support and encouragement, and female sex worker-formed support groups or buddy systems through which they could encourage each other to get tested for HIV. Female sex workers taking PrEP were also described as potentially benefitting from regular, ongoing community education on drug and substance abuse and from population-specific information, education, communication materials. Providing continuous education on the importance of quarterly HIV testing was suggested as potentially beneficial for fishermen taking PrEP.

#### Fears, lack of awareness, and misinformation among target populations

Fear of HIV testing and learning one’s status was cited as a barrier for all groups, both for current HIV-prevention programs and for future efforts to encourage people to get screened for eligibility to take PrEP. Strategies for addressing this fear were continuous community education; creating awareness among the community about the importance of HIV testing and knowing one’s status; organizing moonlight HIV testing; individual counselling; and giving more time to clients in the counselling sessions to help them understand and make decisions about getting tested for HIV and taking PrEP.

Discussants in the serodiscordant couples group said that fears about the side effects of PrEP could deter this population from using it. They suggested that fears could be addressed through continuous community education and sensitization, and by providing examples of cases in which people have used PrEP successfully. Having to disclose HIV test results to a partner was another fear held by this population that could negatively affect recruitment efforts for eligibility screening and ongoing monitoring of HIV status. Strategies the discussants suggested focused on the importance of disclosure and included couples counselling, involving family members in group counselling, and working with peers to have them pass on information regarding the importance of disclosure. Serodiscordant couples were also said to fear the pain of being pricked and losing a lot of blood. Their fears could be allayed, the discussants suggested, by counselling them that the pain is temporary, illustrating the quantity of blood collected, and explaining the reason for the tests. Counselling and community education about blood draws and the importance of sample testing could also help to address widows’ fear of blood draws that might discourage them from getting screened for PrEP eligibility.

Discussants also talked about ignorance and misconceptions as challenges to PrEP implementation. Widows were described as ignorant about HIV-prevention strategies in general; FSWs were said to likely be ignorant and have suspicions about the concept of PrEP; and widows, fishermen, and serodiscordant couples were described as ignorant about blood sample testing, perhaps having the misconception that samples collected for eligibility screening or ongoing monitoring would be sold or used for other purposes. Community education, education to members of the target populations, and individual counselling were suggested as means of creating greater awareness. A strategy to address ignorance and misconceptions among FSWs was group education at the workplace on basic PrEP facts and on the need for HIV testing among PrEP users.

#### Issues for uptake of PrEP among target populations

Other barriers to PrEP implementation were related to uptake and included the distance to the dispensing facilities if centralized (fishermen), the burden of having to take a drug every day (serodiscordant couples), and non-adherence to pill taking and clinic visits (widows, female sex workers, and serodiscordant couples). For drug adherence issues, discussants recommended drug adherence counselling, as well as counselling that involves family members, whereby clients could be asked to have a friend or family member remind them to take the drugs (female sex workers). Also recommended as facilitators for serodiscordant couples were couple counselling and including family members in adherence education. They also recommended collecting locator information for female sex workers to facilitate the process of making home visits intended to encourage adherence to clinic visits.

### Summary

The barriers and facilitators/solutions that discussants provided were frequently population-specific, but there were also broad-level similarities across populations. For example, service delivery barriers to HIV-prevention interventions focused on the need for staff trained specifically to address the needs of particular target populations. Service delivery facilitators to provision of ongoing HIV testing consisted of offering more testing options than just facility-based testing. Stigma was the main community-level barrier for all groups. Barriers at the level of the target populations included the mobility of all four populations; the lifestyle and life circumstances of target populations, especially cultural norms and traditions among fishermen and widows; and fears, lack of awareness, and misinformation among all target populations. Some facilitators and strategies that were proposed by the consultation discussants for addressing these barriers included topic-specific educational efforts within each population and in the community, involvement of partners and family members, mass HIV testing among the general population in order to normalize it, and involvement of peer educators.

## Discussion

We asked consultation discussants to identify barriers and facilitators/solutions to implementing HIV prevention and PrEP interventions—in particular, eligibility screening (via HIV testing and other blood tests) and ongoing HIV testing for PrEP—with fishermen, Luo widows, female sex workers, and HIV serodiscordant couples in Bondo and Rarieda districts, Nyanza province, Kenya. The purpose was to generate tailored strategies that could be used to inform program design for each potential local target population for oral PrEP. We focused on specific target populations for oral PrEP because targeted provision to populations at risk of HIV may maximize oral PrEP’s impact [35-38]. Yet, the utility of these consultation results for some readers may rest on the extent to which they are applicable to other areas of Kenya outside of Bondo and Rarieda districts, as well as contexts outside of Kenya.

On the one hand, many of the barriers to HIV prevention that were identified for the target populations in Bondo and Rarieda (e.g., stigma and fear of learning one’s HIV status) are widespread in sub-Saharan Africa and elsewhere. Many of the barriers also support others’ findings regarding likely challenges to PrEP implementation, particularly stigma and lack of trained staff [39,40].

On the other hand, local tailoring in each country context may be the best approach for developing effective solutions to address these widespread barriers. That said, there will also likely be regional similarities in the applicability of the solutions/facilitators among target populations: fishermen based in the Lake Victoria region in Tanzania and Uganda share characteristics with Kenyan fishermen working in the Bondo area [41-48]; widows in Bondo have commonalities with widows in other countries in sub-Saharan Africa [49-53]; female sex workers in other parts of Kenya and sub-Saharan Africa have similarities with the female sex workers discussed here for Kenya [54-62]; and serodiscordant couples in all regions of sub-Saharan Africa tend to share similar characteristics [63-66]. The four broader groups across sub-Saharan Africa may therefore be affected by the clinical requirements for oral PrEP use in similar ways. Thus, the often innovative solutions and facilitators that consultation discussants provided based on their experience with what has and has not worked with these populations have application for program planning beyond Bondo and Rarieda districts in Kenya. We consider this to be the major strength of the research presented and the utility of the research for international audiences, particularly program planners.

The consultation results also are applicable beyond implementation of oral PrEP, given that regular HIV testing and counselling will be an essential component of any combination prevention strategy [36]. As discussed here, HIV testing and other blood tests (e.g., testing for liver and kidney function) will form part of eligibility screening for oral PrEP, and ongoing HIV testing will be required to address the potential for the acquisition or development of drug-resistant strains of HIV [1-5]. Other ARV-based formulations such as gel, vaginal rings, and injectables will also have, at a minimum, the initial and ongoing HIV testing components, if not the initial lab tests depending on the formulation. Therefore, the consultation results on initial and ongoing HIV testing for oral PrEP will apply to interventions that include these other PrEP formulations as well.

Consultation with local stakeholders in planning for PrEP implementation has been recommended by various researchers and planning groups [38,67,68]. A strength of our consultation is that it engaged local stakeholders who brought real-world, “on the ground” experience to the discussion on oral PrEP. Moreover, their views were communicated to provincial and national public health stakeholders in subsequent consultations, providing an opportunity for their voices to be heard by people who would be involved in high-level planning. In addition, to our knowledge, this consultation is the first activity of its kind—that is, designed to develop practical, locally grounded strategies for PrEP rollout. The expertise and transferrable experience of the community stakeholders with fishermen, widows, female sex workers, and serodiscordant couples will be a valuable contribution to the design of future PrEP interventions in Kenya with these groups.

Our work has several limitations. Originally, our intent in this study was to triangulate the data from this consultation with interview data on uptake of services (i.e., screening for eligibility and ongoing HIV tests), based on the perspectives of members of the target populations. However, we were unable to collect the target populations’ views on the topic due to the early closure of the study after the FEM-PrEP clinical trial was stopped. In addition, collecting the target populations’ perspectives would have added to the credibility of the data in terms of demonstrating support for or divergence from the findings of the consultation. However, the people who participated in the consultation were experts in HIV-prevention interventions among the target populations in this geographical and cultural context, thereby conferring credibility to the findings.

Another limitation is that although the methodology itself—a consultation meeting—was culturally appropriate for collecting the perspectives of these stakeholders, it nonetheless did not yield a transcript that could have been coded qualitatively and that could have supported the written documentation of the consultation. Some detail from the discussions may therefore have remained undocumented.

In addition, although adherence—which has been discussed as a key to the success of any PrEP program [37,69,70]—was brought up briefly by discussants, we did not overtly address it in the consultation. This was because acceptability of taking a pill is likely to be quite different from the “acceptability of a larger package of safety screening, long-term use, behavioral intervention, HIV testing, and potential costs” [68]. Also, we did not specify the type of HIV blood test (finger-prick or serum) to be conducted for initial and ongoing screening, because this was an unknown factor at the time of the research.

We conducted this consultation well before the results of any PrEP trials were available, with the understanding that it would take time to develop and coordinate all the different pieces necessary to mount PrEP interventions, should it prove effective. Such early research was recommended in 2007 [71] and our results are now timely given that “proof of concept” for ARV-based HIV prevention has been shown for oral PrEP and tenofovir-based topical PrEP [35,72-74]. Next steps will be for national and local public health stakeholders in Kenya and countries with similar populations to incorporate the consultation results into demonstration and pilot projects and evaluate the effectiveness of the facilitators and solutions for addressing the barriers. Scale-up of the study results were beyond the scope of this research study; however, in order to facilitate local use of the results, they were formally disseminated to national-level public health stakeholders both at a national meeting on ARV-based HIV prevention in Naivasha, Kenya in 2012 and in a report summarizing the overall study findings in 2013. It is now up to public health program planners at the national and provincial levels to implement the research results as they see fit.

## Conclusions

The clinical protocols that have been recommended by CDC and WHO for determining PrEP eligibility and conducting ongoing HIV and safety testing are likely to be applied in a fairly universal way across different country settings and populations. However, strategies for encouraging people to get screened for eligibility and to comply with the ongoing HIV testing schedules will clearly have to be tailored for the local context to meet population-specific needs. Our results nonetheless have application for other, similar target populations in the region and reach beyond oral PrEP to other ARV-based formulations for PrEP of HIV.

## Endnote

^a^Note that the consultation was held before the announcement of results from any oral PrEP clinical trials.

## Abbreviations

PrEP: Pre-exposure prophylaxis; Note: FEM-PrEP is not an acronym.

## Competing interests

The authors declare that they have no competing interests.

## Authors’ contributions

NM designed the study, led design of the consultation reported on, conducted data analysis, and led the drafting of the manuscript. JO contributed to the consultation design, conducted the consultation and collected the data, wrote up the consultation results on which the results section is based, verified the analyzed data, and revised the draft critically. CW contributed to the consultation design and results write-up, and revised the draft critically. KA contributed to the consultation design, conducted the consultation and collected the data, reviewed the consultation results write-up critically, verified the analyzed data, and revised the draft critically. All authors read and approved the final manuscript.

## Pre-publication history

The pre-publication history for this paper can be accessed here:

http://www.biomedcentral.com/1472-6963/14/231/prepub
